# Food Marketing in Facebook to Thai Children and Youth: An Assessment of the Efficacy of Thai Regulations

**DOI:** 10.3390/ijerph16071204

**Published:** 2019-04-03

**Authors:** Nongnuch Jaichuen, Vuthiphan Vongmongkol, Rapeepong Suphanchaimat, Nonglapat Sasiwatpaisit, Viroj Tangcharoensathien

**Affiliations:** 1 International Health Policy Program (IHPP), The Ministry of Public Health, Nonthaburi 11000, Thailand; vuthiphan@ihpp.thaigov.net (V.V.); rapeepong@ihpp.thaigov.net (R.S.); nonglapat@ihpp.thaigov.net (N.S.); viroj@ihpp.thaigov.net (V.T.); 2 Bureau of Epidemiology, Department of Disease Control, The Ministry of Public Health, Nonthaburi 11000, Thailand

**Keywords:** marketing, food, children and youth, Facebook, Thailand

## Abstract

To assess the marketing of food on Facebook in relation to Government regulations and the industry’s self-regulatory codes in Thailand, Facebook pages of 30 of the most popular food brands with young people in Thailand and consumer engagement (number of likes, shares, and comments) were recorded and had their marketing content transcribed during the month of December 2017. We coded the contents into 17 marketing techniques and conducted content analysis of these posts in relation to Government regulations and the industry’s self-regulatory codes. A total 752 posts were identified in one month. Retail food brand pages had the highest figures for engagement by Facebook viewers. The most common marketing techniques were the use of pictures (632 posts), followed by branding elements (569 posts) and hashtags (438 posts). Out of a total of 228 spot advert posts, all confectionery adverts and almost all (99.5%) soft drink adverts did not display the advertising license number and none of the confectionery adverts displayed the warning messages as required by law. Confectionery, retail food, and soft drink advertising violated the industry’s self-regulatory codes. The food brand Facebook pages in Thailand do not comply with Government regulations and the industry’s self-regulatory codes. The Government, civil society organizations, and academia should monitor these violations and improve enforcement.

## 1. Introduction

At least 2.8 million global deaths were attributable to overweight or obesity in 2010 [1]. Globally, the prevalence of obesity has nearly tripled since 1975 [2]. In 2014 worldwide, 11% of men and 15% of women over 18 years old were obese. An estimated 42 million children under five were overweight in 2013 [3]. In 2014, the Thailand national health examination survey reported 37.5% obesity in Thai adults over 15 years. In Thai men, the prevalence of adult obesity rose from 28.4% in 2009 to 32.9% and in Thai women from 40.7% in to 41.8% in the same period [4]. Also in 2014, 13.9% of Thai children between six and 14 years old were overweight or obese [5].

The food environment, including marketing of unhealthy food, contributes to overweight and obesity [6,7]. Marketing unhealthy food through digital media such as SMS and email induces unhealthy food consumption in children and youths [8,9] and creates product loyalty [10]. Many media channels exist in Thailand, including television, radio, and billboard; however, Facebook is the most popular media with the highest proportion of users nationwide. One third of total food advertising expenditure in Thailand is through Facebook, at 129 million US$. The nonalcoholic beverages manufacturers in Thailand spend 664 million baht per year on digital advertising [11]. In Thailand, 89% of households own a computer [12] and 75.5% of the population has access to the internet [13]. Moreover, the 2017 national internet survey found that 99.6% of Thai people with internet access use Facebook and this figure was 90% among youths [14]. As a result, food industries maximize use of the Facebook to create brand and product loyalty among adolescents and young people [15].

Evidence in Australia indicates that the majority of food and nonalcoholic beverage marketing online is to promote unhealthy and obesogenic products [16,17]. Food marketing on Facebook frequently uses tactics such as indirect product association, engagement technique, branding, user-generated content, and interactive games and applications (apps) [16,17]. There are limitations of self-regulatory codes to control marketing strategies in the context of new media [14]. Prior studies in Thailand have reported the associations between information exposure and satisfaction and brand loyalty among Facebook members [18].

### 1.1. Government Regulation

The Government has three regulations for food marketing. Firstly, the 2016 Notification of the Ministry of Public Health (B.E. 2559) requires that foods such as fried or baked potatoes, fried or baked corn, extruded snacks, crackers, biscuits, wafers, chocolate, instant food, and frozen food have printed package labelling with the following warning messages: “Eat moderately and exercise for good health”, using clear and bold Thai fonts. Also, advertising these food products through media must have a warning message with clear sound and wording for at least five seconds [19]. Secondly, the Fifth Ministerial Regulation 1991 (B.E. 2534) issued under the Consumer Protection Act 1979 (B.E. 2522) requires that market promotions such as a special price, vouchers, competitions, offers, rebates, and sweepstakes with a grand prize must display detailed information and conditions [20]. The fine for violations of these regulations ranges from 5000 to 100,000 baht [21,22].

The aim of these regulations is to reduce exposure to and the persuasive power of unhealthy food and beverage marketing. These regulations control advertising which is released through broadcasting and radio television, image display, films, or newspaper or other printed materials, or social media, or any other method [19,20]. Thai Food and Drug Administration and office of the Consumer Protection Board are responsible for ensuring these regulations are complied with and enforcing the fines.

### 1.2. The Industry’s Self-Regulatory Codes

The food and beverage industry and the advertising industry has some self-regulatory codes undertaken on a voluntary basis; for example, the Code for Food and Snack Advertising to Children [23], the International Chamber of Commerce (ICC) Framework for Responsible Food and Beverage Marketing Communications 2012 [24], the Code for Advertising and Marketing Communications to Children [25], the Food & Beverages Advertising and Marketing Communications Code [26], and the Self-Regulatory Program for Children’s Advertising [27].

The main contents are summarized in Box 1. Among others, the self-regulatory codes prohibit the promotion of inappropriate consumption, putting pressure on consumers to purchase the products, using sexualization where individuals are regarded as sex objects and evaluated in terms of their physical characteristics and sexiness, and the use of popular personalities in all media. The international codes apply to advertisements targeting Thai children and youth.

Box 1 **Summary of the main contents in the industry’s self-regulatory codes.**

1. **The promotion of inappropriate consumption**; the food industry should not encourage excessive consumption of food products or snacks instead of main meals, avoid encouraging poor nutritional habits or an unhealthy lifestyle in children, must neither encourage nor promote an inactive lifestyle or unhealthy eating or drinking habits, it will not persuade children to force their parents or other to purchase the advertised products
2. **Putting pressure on consumers to purchase the products**; the food industry refrains from sending messages which make children feel inferior to others if their parents or others do not buy these products or services, messages must not imply that possession or use of a particular food and beverage product will result in physical, social, or psychological advantage over other children who do not consume these products; and do not exaggerate their products by using words such as “only” or “just”.
3. **Using sexualization**; industry will refrain from using individuals (in particular, the presenters) as sex objects evaluated in terms of their physical characteristics and their sexiness;
4. **The use of popular personalities**; Industry will refrain from using popular personalities or celebrities (either live or animated) to advertise or market their products which obscures the distinction between commercial promotions and related program content.

Evidence shows that advertising, sponsorship, product placement, sales promotion, cross-promotions, using celebrities, brand mascots, or characters popular with children, web sites, packaging, labelling, and point-of-purchase displays, e-mails and text messages, philanthropic activities tied to branding opportunities, and communication through digital media, viral marketing and by word of-mouth influences children’s food preferences, purchase requests, and consumption patterns [28,29]. There is limited evidence on the techniques of unhealthy food marketing to children and youth. Food marketing on Facebook is not assessed against government statutory regulations and industry self-regulatory code in Thailand. Monitoring the nature and extent of food marketing in Facebook is essential to provide information on the degree of this issue to support policy action. As Facebook is so widely used, this study quantified the magnitude and profile of contents of food marketing on Facebook to children and youth, and assessed the contents to see if they complied with Government regulations and Thailand industry’s self-regulatory codes.

## 2. Materials and Methods

Based on the methods of Freeman [16], this study describes food marketing techniques and tactics on Facebook including using pictures, branding elements, hash tags, conversations, special promotions, links, videos, competitions, prizes, give-aways, branded characters, celebrities, games, apps, and several others.

A quantitative descriptive survey was applied to collect the marketing contents of the most popular food brands on Facebook pages and we analyzed the contents to see if they comply with the Government regulation and self-regulatory codes.

### 2.1. Population and Sample

This study covered three groups of food and beverage: confectionery, soft drinks, and retail food according to the Socialbakers’ categorization which had the greatest number of Thai users who “liked” the page, using data from the social media monitoring site called Socialbakers [30]. Confectionery was classified in two subgroups of (1) baked products, biscuits, and snack bars; (2) confectionery, ice cream, and frozen desserts. Soft drink was defined as nonalcoholic beverages and further categorized as (1) green tea; (2) carbonated beverages; (3) energy drink. We defined retail food as chain-restaurant food.

On 1 December 2017 we selected thirty Facebook pages of food and beverage brands in Thailand. These comprised the top ten confectionery Facebook pages, the top ten soft drinks pages, and the top ten retail food pages. This list was publicly available, with all data extracted directly from Facebook. The list was limited to Thai-based brand pages which were popular among Thai users, and excluded international Facebook pages.

### 2.2. Data Coding

We adapted a content coding tool from previously validated study instruments used to assess unhealthy food and beverage in television advertising by the authors [31]. See Appendix A Table A1, Table A2 and Table A3 for detail. To assess whether marketing strategies had complied with Government regulations and the industry’s self-regulatory code, we then developed a coding tool against the contents of these regulations. See Box 1.

### 2.3. Data Collection

Pilot test: a single researcher collected and coded the content to assess its appropriateness and completeness and determine which data would be collected from the page. Thirty food brand pages were recorded for 24 h, every day for the whole month of December 2017. Each page was examined every hour. All data were collected using a screen grab from each page of the Facebook timeline and saved as PDF files for content analysis.

All food brands were coded in Microsoft Excel. The following data was recorded for each post: page name, date of recording, company name, food product category, date of page launch, number of people talking about the page, number of page members, number of likes, number of shares, number of posts by page only, marketing techniques, Government regulations, and self-regulations.

There are three steps of data collection; data collection, data coding, and coding interpretation. In the first process, data collection was done by three researchers (Nongnuch Jaichuen (N.J.), V.V., N.S.). In the second process, data coding was done by one researcher (N.J.). In the third process, coding interpretation was done by three independent researchers (N.J., V.V., N.S.). Then, the codes were cross-checked by another researcher. Any disagreements were settled by discussion and consensus.

### 2.4. Data Analysis

There are four main analyses: (1) profile and timeline posts of food Facebook brand pages, (2) marketing techniques, (3) compliance with Government regulations, and (4) compliance with the industry’s self-regulatory codes. In the first analysis, the numbers of fan pages and like or reactions were assessed by descriptive statistics including frequency, means, standard deviation, and median. For the second to fourth analyses, frequencies and percentage were used to describe the food marketing by food categories, their marketing techniques, and compliance with Government regulations and self-regulatory codes. We use SPSS for Windows version 18.0 (SPSS Inc., Chicago, IL, USA.) for statistical analysis.

## 3. Results

The most popular food Facebook brand page out of the thirty most popular in Thailand, according to number of users, was Kentucky Fried Chicken (KFC) Thailand, with a total of 3,871,239 fans in December 2017. The 30th most popular page was 100Plus Thailand (soft drink) with 254,698 members. The most active page was Pizza Hut. It had 70 posts in one month. KFC had the highest number of likes for its posts in a one-month period at 141,195, while KFC Thailand had the highest average number of 3085.8 likes per post. Wall’s Thailand (ice cream) had the highest average number of messages shared at 506.5 per post, and Wall’s Thailand attracted the highest average number of comments, at 414.8 per post. In total, the retail food administrator of the Facebook page contributed to the highest numbers of posts in December 2017 (31,781 posts), followed by soft drinks (4626 posts) and confectionery (2461 posts) (Table 1 and Table 2).

### 3.1. Marketing Techniques

The most common techniques applied in Facebook marketing were the use of pictures (632 posts), followed by branding elements (569 posts) and hashtags (438 posts). The majority of images were pictures of food and beverage products. A hashtag is a word or phrase preceded by a hash mark # which is used within a message as a keyword to advertise a product and facilitate a search. Analysis of marketing techniques by food categories showed that out of a total of 524 posts, the retail food group used pictures in 90.5% of their posts. Confectionery and soft drink companies applied branding elements in 90.9% and 90.8% of their total 44 and 184 posts, respectively. This study did not find the use of vouchers, offers, or rebates in marketing techniques on Facebook. Facebook used these marketing techniques appealing to fans to help promote positive attitudes towards the brand and products, potentially making them more familiar with and develop loyalty to the brand and products (Table 3).

### 3.2. Compliance with Government Regulations

When advertising strategies on Facebook were assessed against three Government regulations, this study indicated that none or a very small proportion of food Facebook brand pages had complied. Of these, 100% of confectionery and 99.5% of soft drink pages did not display an advertising license number. None of the confectionery posts (44) displayed warning messages, 81.8% of retail food posts (524), 81.8% of soft drink posts (184), and 80% of confectionery posts (44) had incompletely-displayed information and conditions of sweepstakes (Table 4).

### 3.3. Compliance with Industry’s Self-Regulatory Codes

When Facebook marketing strategies were assessed against the industry’s self-regulatory codes, we found that none of the food Facebook brand pages conformed with these voluntary codes. Confectionery displayed messages which encouraged and induced excessive consumption of their products; while retail food used sexualization and the word “only” or “just” to exaggerate the value of their products. One of the uploaded images by a retail food company depicted a famous Thai male singer embraced by a young male seller wearing a brand dress; where the conversation text between the two presenters related to sexualization. Soft drink adverts used popular personalities in marketing their products (Table 5).

## 4. Discussion

Facebook is one of the most popular social media channels for children and adolescents in Thailand; this prompted us to assess compliance to Government regulation and self-regulatory codes. Retail food brand Facebook pages were the most popular, and had the highest numbers of posts and likes, the highest average number of likes per post, sharing messages to others, and comments.

The most common marketing technique on Facebook was the use of pictures, followed by branding elements and hashtags. Retail food pages used pictures more than other techniques, while confectionery and soft drink applied branding elements as their main technique. The retail food administrators preferred to apply posts focusing on price reductions and conversations, which better interact with consumers than the confectionery group. Focusing on price competition and conversation by the retail food group can be an effective strategy to boost sales volume. The nature of cooked and ready to eat food items, such as pizza and burgers, requires effective prices reduction to customer, hence Facebook is used as a key channel. A common trend emerged that food and nonalcoholic beverage websites in high and middle income countries used pictures and brand elements to promote their products and increase their brand loyalty [32,33]. However, one of the popular marketing techniques in Thailand is the use of hashtags because this technique can amplify opportunities to carry messages about products, campaigns, and events in order to reach large numbers of children and adolescents [15,34,35,36,37].

None of the food and nonalcoholic beverage Facebook brand pages complied with Government regulations or the industry’s self-regulatory codes of practice. The Food Act B.E. 2522 (1979) prescribes that before an advertisement is released through broadcasting and radio television, image display, films, newspapers, other printed materials, or any other methods, the advertising content must be reviewed by an authorized person before permission to advertise is granted. After permission, advertising must display the advertising license number. Without permission, an advertisement cannot be released [19]. Our findings showed that confectionery and soft drink did not display the advertising license number. In addition, the Ministerial Notification (B.E. 2550) (2007) by the Ministry of Public Health on “Labeling of Certain Pre-cooked Ready-to-eat Food” stated that ready-to-eat food and extruded snack advertisements must display text or voice messages stating “Consume little and exercise for good health” [19] but none of the confectionery food displayed such warning messages. Violation of regulations by the food industry could partly occur as a result of the lack of regulatory capacities in surveillance and taking action on noncompliance. Moreover, the Ministry of Public Health (MOPH) Ministerial Notification (B.E. 2550) (2007) did not apply obesogenic food such as sugar-sweetened drinks and energy-dense retail food.

The Ministerial Regulation B.E. 2534 (1991) issued under the Consumer Protection Act B.E. 2522 (1979) prescribed that advertisements containing special price promotions, vouchers, offers, rebates and sweepstakes, competitions, and prizes shall display conditions such as the terms of promotion, and the starting and closing dates [20]. Unfortunately, this regulation failed to enforce the contents of marketing promotions on Facebook.

The Advertising Association of Thailand was responsible for issuing the Code for Food and Snack Advertising to Children [23]. However, our findings showed full violations of these voluntary codes. Without a responsible agency to oversee and monitor adherence to the code, the current self-regulatory mechanisms were not effective in protecting children and adolescents, who were minors, from excessive marketing promotion of food and beverages. 

Findings from this study were consistent with previous international studies on poor compliance with self-regulatory codes applied by the food, alcohol, and tobacco industries using digital media [38,39,40,41]. It should be noted that the Thai code prohibited the use of “only” or “just” which exaggerated the value or superiority of their products, and the use of sexualization. The contents of the Thai code were similar to the international codes of self-regulations in Australia, United States of America, and the International Chamber of Commerce (ICC) [24,25,26,27].

This study has certain limitations; it assesses the contents of Facebook media advertising but does not examine the actual exposure of children and youth to these marketing tactics. This study cannot assess the age and profile of consumers who have access to these Facebook brand pages. However, the national survey confirmed that 63% of children between 6 and 14 years, and 90% of youth between 15 and 24 years had been exposed to Facebook in 2017 [14]. In addition, Thai adolescents who are 17 years or younger spend their free time surfing Facebook for at least three hours and thirty minutes per day [42]. Regarding research methodology, there are some concerning points. For instance, inter-rater reliability statistic was not tested because there was only one researcher taking care of the coding process. However, this might not affect the data reliability much as all codes were checked by another researcher after the coding was completed. Moreover, this study provided only a snapshot picture of Facebook food marketing in Thailand. As we all know, the food marketing situation is hugely dynamic due to various uncontrolled contextual environments, and the study findings may not reflect the most up-to-date status of food marketing in social media in Thailand.

## 5. Conclusions

Brands are using the interactive and social aspects of Facebook to market their products. Results from this study show that food Facebook brand pages in Thailand do not comply with Government regulations and the food and beverages industry’s self-regulatory codes. Though Thailand has several laws and regulations to restrict marketing and advertising, in particular to children and young adolescents, the loopholes identified by this study prompt the need for policies to closely monitor and enforce regulation on inappropriate marketing and advertising of soft drinks and retail food. The current regulations and guidelines must updated in line with new evidence and loopholes identified by this study and strengthened to ensure these regulations cover all forms of food and beverage advertising to children. The government, nongovernment organizations, such as the consumer protection foundation groups, and research agencies should establish an effective monitoring system and demand that government regulators take serious actions on violations in order to protect the rights and health of Thai children and youth in the context of challenges from increased prevalence of obesity and noncommunicable diseases.

## Figures and Tables

**Table 1:** Profiles of the 30 most popular food Facebook pages in Thailand, 1–31 December 2017.

Name	Date Page Launched	Number of Fans			Number of Talking About
		1 December 2017	31 December 2017	Increased/Decreased	
**Confectionery**					
Lays Thailand	2011	1,034,468	1,035,647	1179	36,333
Magnum Thailand	May 2012	942,483	942,774	291	23,531
Cornetto Thailand	2010	798,513	800,025	1512	16,600
Glico Thailand	April 2011	610,134	611,670	1536	-
Wall’s Thailand	1 April 2013	494,670	522,061	27,391	90,849
KitKat	August 2011	400,208	403,528	3320	2204
Voiz Thailand	-	387,940	402,920	14,980	-
Twisties Cheetos	January 2010	367,202	366,536	−666	130
Glico ice TH	June 2015	294,278	294,107	−171	-
Nestle Ice Cream TH	-	276,608	276,136	−472	11,460
**Average confectionery**		**560,650**	**565,540**	**4890**	**18,111.0**
**Soft Drink**					
Oishi Drink Station	-	3,730,806	3,750,579	19,773	-
ICHITAN	-	3,602,376	3,595,839	−6537	-
Coca-Cola	-	2,787,215	2,792,670	5455	83,892
PepsiThai	2012	2,698,849	2,703,217	4368	23,636
Big Cola	-	811,545	811,735	190	-
Est	2012	647,047	672,631	25,584	153,864
Puriku	-	432,145	431,047	−1098	-
Sponsor	2010	420,429	420,326	−103	-
Fanta	-	387,973	387,765	−208	7125
100PlusThailand	2011	255,299	254,698	−601	2566
**Average soft drink**		**1,577,368**	**1,582,051**	**4682**	**27,108.3**
**Retail Food**					
KFC	November 2010	3,794,927	3,871,239	76,312	12,908
McDonald’s	August 2009	1,643,856	1,655,452	11,596	50,764
Starbucks Thailand	-	1,294,560	1,316,415	21,855	77,753
SizzlerThai	-	1,275,627	1,279,325	3698	-
The Pizza Company 1112 Lovers	-	1,100,163	1,132,728	32,565	-
Burger King Thailand	2011	1,088,222	1,092,435	4213	6
Pizza Hut	2010	1,066,988	1,091,658	24,670	28,758
Dairy Queen Thailand	-	1,042,777	1,047,745	4968	50,608
We Love Swensen’s	December 2011	1,011,474	1,041,597	30,123	-
Hot Pot Buffet	-	923,342	935,645	12,303	-
**Average retail food**		**1,424,194**	**1,446,424**	**22,230**	**22,079.7**

Note. - data is not available.

**Table 2:** Timeline posts by page of food Facebook pages 1–31 December 2017.

Name	Number of Posts by Page Only	Number of Likes or Reactions (Total for All Posts)	Number of Shares (Total for All Posts)	Number of Comments (Total for All Posts)	Mean (SD) Likes Reactions per Post	Mean (SD) Shares per Post	Mean (SD) Comments per Post
**Confectionery**							
Lays Thailand	2	130	0	5	65 (22.6)	0	2.5 (3.5)
Magnum Thailand	0	0	0	0	0	0	0
Cornetto Thailand	0	0	0	0	0	0	0
Glico Thailand	16	1665	150	245	123.5 (256.2)	9.4 (20.9)	15.3 (31.2)
Wall’s Thailand	4	2215	2026	1659	531.3 (420.0)	506.5 (415.1)	414.8 (343.7)
KitKat	4	123	3	12	61.5 (57.2)	1.5 (2.1)	6.0 (5.7)
Voiz Thailand	11	4221	137	96	383.7 (811.3)	12.5 (26.0)	8.73 (21.2)
Twisties Cheetos	0	0	0	0	0	0	0
Glico ice TH	6	1290	181	270	215.0 (389.1)	30.17 (34.2)	45.0 (87.6)
Nestle Ice Cream TH	1	9	0	0	9	0	0
**Total of confectionery**	**44**	**9653**	**2497**	**2287**	**235.1 (489.5)**	**59.5 (186.3)**	**54.5 (155.6)**
**Soft Drink**							
Oishi Drink Station	23	5248	1242	1277	228.2 (273.2)	54.0 (90.5)	55.5 (145.9)
ICHITAN	22	6571	1203	1099	298.7 (267.2)	54.7 (155.8)	50.0 (149.7)
Coca-Cola	6	968	449	207	161.4 (201.3)	74.8 (85.9)	34.5 (46.5)
PepsiThai	19	2850	86	129	150.0 (108.0)	4.5 (9.8)	6.8 (18.5)
Big Cola	23	911	12	61	39.6 (26.7)	0.5 (0.6)	2.7 (6.0)
Est	16	8259	1468	744	516.2 (322.5)	91.8 (116.2)	46.5 (109.8)
Puriku	57	6161	287	688	108.1 (125.9)	5.1 (10.7)	12.1 (41.8)
Sponsor	18	1272	275	421	70.7 (158.7)	15.5 (30.7)	24.5 (70.3)
Fanta	0	0	0	0	0	0	0
100PlusThailand	0	0	0	0	0	0	0
**Total of soft drink**	**184**	**32,240**	**5022**	**4626**	**175.2 (228.9)**	**27.3 (78.4)**	**25.1 (87.2)**
**Retail Food**							
KFC	46	141,195	7794	8110	3085.8 (4985.6)	169.4 (506.0)	176.3 (432.8)
McDonald’s	68	28,916	1167	1821	425.2 (939.1)	17.2 (31.6)	26.8 (59.9)
Starbucks Thailand	25	33,830	2051	1597	1353.2 (1398.0)	80.6 (159.8)	63.9 (116.6)
SizzlerThai	64	49,005	795	1969	765.7 (478.4)	12.4 (22.9)	30.8 (29.4)
The Pizza Company 1112 Lovers	45	13,048	2262	3932	289.9 (631.1)	50.3 (136.3)	87.38 (167.1)
Burger King Thailand	66	15,944	954	1137	241.6 (178.4)	14.5 (69.3)	17.2 (78.4)
Pizza Hut	70	25,678	3810	5188	366.8 (392.2)	54.4 (115.7)	74.1 (122.4)
Dairy Queen Thailand	33	5773	401	292	174.9 (85.1)	12.2 (12.1)	8.9 (16.8)
We Love Swensen’s	42	15,923	4194	4179	379.1 (493.8)	99.9 (187.9)	99.5 (187.1)
Hot Pot Buffet	65	20,045	2368	3556	308.4 (307.6)	36.4 (88.9)	54.7 (100.8)
**Total of retail food**	**524**	**349,357**	**25,796**	**31,781**	**1757.5 (668.1)**	**182.1 (49.2)**	**168.7 (60.7)**
**Overall**	**752**	**391,250**	**33,315**	**38,694**	**523.0 (1493.9)**	**44.4 (163.2)**	**51.6 (152.6)**

**Table 3:** Marketing techniques used by the Facebook pages, 1–31 December 2017.

Food Categories	Number of Posts	Marketing Techniques, *n* (%)																
		Pictures	Branding Elements	Hashtag *	Conversations	Special Price Promotions	Links	Videos	Competition, Prizes, Giveaways	Branded Characters	Celebrities	Games	Apps	Sponsorships and Partnerships	Quizzes and Polls	Corporate Social Responsibility and Philanthropy	Sport People	Vouchers, Offers, Rebates
Confectionery	44	31 (70.5)	40 (90.9)	18 (40.9)	23 (52.3)	4 (9.1)	2 (4.5)	12 (27.3)	5 (11.4)	5 (11.4)	2 (4.5)	2 (4.5)	1 (2.3)	0 (0.0)	0 (0.0)	1 (2.3)	2 (4.5)	0 (0.0)
Soft drink	184	125 (67.9	167 (90.8)	132 (71.7)	68 (37.0)	6 (3.3)	28 (15.2)	56 (30.4)	22 (12.0)	16 (8.7)	25 (13.6)	16 (8.7)	19 (10.3)	6 (3.3)	2 (1.1)	1 (0.5)	0 (0.0)	0 (0.0)
Retail food	524	476 (90.5)	362 (68.8)	288 (54.8)	267 (50.8)	210 (39.9)	168 (31.9)	60 (11.4)	35 (6.7)	21 (4.0)	4 (0.8)	9 (1.7)	2 (0.4)	0 (0.0)	2 (0.4)	1 (12.4)	0 (0.0)	0 (0.0)
Overall	752	632 (83.8)	569 (75.5)	438 (58.1)	358 (47.5)	220 (29.2)	198 (26.3)	128 (17.0)	62 (8.2)	42 (5.6)	31 (4.1)	27 (3.6)	22 (2.9)	6 (0.8)	4 (0.5)	3 (0.4)	2 (0.3)	0 (0.0)

Note. One post consists of more than one technique. * New marketing techniques identified specific to Thai context.

**Table 4:** Compliance with three government statutory regulations.

Food Categories	Number of Posts	Contents, *n* (%)							
		1. Display License Number in Spot Ads (*n* = 228)		2. Should Read “Warning Massage” (*n* = 33)		3. Promotion (*n* = 282)			
						3.1 Special Price Promotions (*n* = 220)		3.2 Sweepstakes (*n* = 62)	
		Yes	No	Yes	No	Yes	No	Yes	No
Confectionery	44	0 (0.0)	44 (100.0)	0 (0.0)	33 (100.0)	2 (50.0)	2 (50.0)	1 (20.0)	4 (80.0)
Soft drink	184	1 (0.5)	183 (99.5)	-	-	2 (33.3)	4 (66.7)	4 (18.2)	18 (81.8)
Retail food	524	-	-	-	-	91 (45.5)	119 (59.5)	6 (17.1)	29 (82.9)

Note: - means that the government statutory regulations do not control the soft drink and retail food group on spot advertising and warning message.

**Table 5:** Compliance with industry’s self-regulatory codes of conduct.

Food Category	Number of Posts	Contents, *n* (%)							
		1. Promote Inappropriate Consumption (*n* = 27)		2. Pressure to Purchase (*n* = 99)		3. Sexualisation (*n* = 3)		4. Popular Personalities (*n* = 33)	
		Yes	No	Yes	No	Yes	No	Yes	No
Confectionery	44	0 (0.0)	2 (100.0)	-	-	-	-	0 (0.0)	4 (100.0)
Soft drink	184	0 (0.0)	1 (100.0)	0 (0.0)	4 (100.0)	0 (0.0)	1 (100.0)	0 (0.0)	25 (100.0)
Retail food	524	0 (0.0)	24 (100.0)	0 (0.0)	95 (100.0)	0 (0.0)	2 (100.0)	0 (0.0)	4 (100.0)

## Appendix A

**Table A1:** Overview of government statutory regulations.

No.	Government Statutory Regulations	Content	Summary
1	Foods Act, B.E.2522 (1979)	Before an advertisement is released through broadcasting and radio television, image display, films, or newspaper or other printed materials, or any other method, the advertisement must be checked and reviewed by an authorized person who may grant permission. Without permission, an advertisement cannot be released.	Spot advertising must display the advertising license number
2	Notification of the Ministry of Public Health, B.E.2559 (2016) regarding Labeling of Certain Pre-cooked Ready-to-eat Food	Requires fried or baked potatoes, fried or baked corn, extruded snack, cracker, biscuit, wafer, chocolate, ice cream, packaged food and instant food to produce package labeling and to advertise with the following warning messages “Eat moderately and exercise for good health” using clear and bold text.	Warning messages “Eat moderately and exercise for good health” must be displayed or released in any advertising (either in a print or in voice, as appropriate).
3	The 2008 Food and Drug Administration Notification on Rules on Advertising Foods (B.E.2551)	Requires that any presenter for instant gelatin or jelly targeting children, must be older than three years. The age of the presenter advertising instant gelatin or jelly containing glucomannan or glucomannan flour must be older than 12 years	the age of presenter for instant gelatin or jelly and instant gelatin or jelly containing glucomannan or glucomannan flour
4	The Fifth Ministerial Regulation, B.E.2534 (1991) under Consumer Protection Act, B.E.2522 (1979)	Special price promotions, vouchers, offers, rebates and sweepstakes, competition, prizes which are permitted to advertising must be identified information and condition.	Promotions
4.1	Special price promotions, vouchers, offers, rebates	An advertising which includes special price promotions, vouchers, offers, or rebates must (1) contain a summary of the basic rules for the sweepstake, competition, prizes; (2) clearly include the opening and closing date for entries; (3) inform type and value of gift or premium; (4) inform the place to receive gift or premium	Special price promotions, vouchers, offers, or rebates must be displayed information and condition.
4.2	Sweepstakes, competition, prizes	An advertising which includes sweepstakes, competition, prizes must (1) contain a summary of the basic rules for the sweepstakes, competition, prizes; (2) clearly include the opening and closing date for entries; (3) inform type and value of prizes or rewards; (4) announce the winner via media	Sweepstakes, competition, or prizes must be displayed information and condition.

**Table A2:** Overview of industry self-regulatory codes.

No.	Content	Advertising Association of Thailand (AAT) ^1^	International Chamber of Commerce (ICC) ^2^	Australian Association of National Advertisers (AANA) ^3^	Children’s Advertising Review Unit (CARU) ^4^
**0**	**Definition of children**	0–12 years old	0–12 years old	0–14 years old	0–12 years old
1	**Promote inappropriate consumption**				
1.1	Advertisements or marketing communications must not encourage or condone excessive consumption of any food.	/	/	/	/
1.2	Advertising or marketing communications to children shall not encourage consuming snack foods instead of main meals.	/	/	-	/
1.3	Advertisements must avoid any thing likely to encourage poor nutritional habits or an unhealthy lifestyle among children.	-	-	-	/
1.4	Advertising or marketing communications to children on food or beverages must neither encourage nor promote an inactive lifestyle or unhealthy eating or drinking habits.	-	/	/	/
2	**Pressure to purchase**	-	-	-	
2.1	Advertisements or marketing communications should not include any direct appeal to children which persuades their parents or other adults to buy the advertised products for them.	/	/	/	/
2.2	Advertisements or marketing communications must not imply that children will be inferior to others, disloyal or will have let someone down, if they or their family do not buy, consume or use a product or service	-	/	/	-
2.3	Advertising or marketing communications to children shall not state nor imply that possession or use of a particular children’s food or beverage product will afford physical, social or psychological advantage over other children, or that non possession of the children’s food or beverage product would have the opposite effects.	-	/	/	-
2.4	Prices, if mentioned in advertising or Marketing Communications to children, must be accurately presented in a way which can be clearly understood by children and must not be exaggerated by certain words such as “only” or “just”.	-	/	/	-
3	**Sexualization** Advertising or marketing communications to children must not include sexual imagery in contravention of prevailing community standards and state or imply that children are sexual beings and that ownership or enjoyment of a Product will enhance their sexuality.	-	-	/	/
4	**Popular Personalities** Advertising or marketing communications to children must not use popular personalities or celebrities (live or animated) to advertise or market products or premiums in a manner that obscures the distinction between commercial promotions and program or editorial content.	-	-	/	-

^1^ Code for Food and Snack Advertising to Children, the Advertising Association of Thailand (AAT), Thailand; ^2^ ICC Framework for Responsible Food and Beverage Marketing Communications 2012, International Chamber of Commerce (ICC); ^3^ Code for Advertising & Marketing Communications to Children and Food & Beverages Advertising & Marketing Communications Code, the Australian Association of National Advertisers (AANA), Australia; ^4^ Self-Regulatory Program for Children’s Advertising, the Children’s Advertising Review Unit (CARU), the United States of America.

**Table A3:** Definition of marketing techniques.

Marketing Techniques	Definition
1. Pictures	Digital images of the product, users, and promotional events
2. Branding elements	Any logos, colors, trademarks, or slogans
3. Hash tags	mark # which is used within a message as a keyword to advertise a product and facilitate a search
4. Conversations	The page administrator responds to page member posts and comments and shares member content with other members.
5. Special promotions	Limited-time offers, discount menus, 2 for 1 deals, or other reduced-price advertisements
6. Links	Any page posts that include a link to an external page or additional content not found within the Facebook page
7. Videos	Can either be posted directly to Facebook or linked through YouTube
8. Competitions, prizes, give-aways	Any contest involving a participant entry, including minimal requirements such as simply liking a post; giveaways also include free product samples and other items with purchase.
9. Vouchers, offers, rebates	Includes those that consumers print off or for which they enter an electronic code; offers are specific to Facebook and made exclusively available to those who like the page.
10. Branded characters	Any characters featured on the page developed by the brand
11. Celebrities	People with an entertainment or media profile excluding sport people
12. Sport people	Any person (adult or child) profiled for their athletic or sporting achievements
13. Conversations	The page administrator responds to page member posts and comments and shares member content with other members.
14. Games	Interactive and entertaining applications that feature the brand
15. Quizzes and Polls	Can be embedded directly into the Facebook timeline; they are a feature available to all brand pages as a way of encouraging participation and interaction.
16. Apps	Both links to any smartphone apps and any apps embedded in the Facebook page. Facebook allows page administrators to develop a variety of application tabs on their pages, including retail store location finders, other social media channel feeds, ordering platforms, feedback, and promotional offers.
17. Sponsorships and partnership	Any events that the brand supports or other brands or services the brand partners with, excluding charitable organizations
18. Corporate social responsibility and philanthropy	Promotion of any ethical or sustainable initiative or charitable work undertaken by the brand
